# Spatial distribution of residential environment, genetic susceptibility, and psoriasis: A prospective cohort study

**DOI:** 10.7189/jogh.14.04139

**Published:** 2024-08-06

**Authors:** Li Chen, Huimin Chen, Li Mo, Min He, Ying Zhao, Tianqi Tan, Ping Yao, Yuhan Tang, Xiangzi Li, Yanyan Li

**Affiliations:** 1 Shenzhen Center for Chronic Disease Control, Shenzhen Institute of Dermatology, Shenzhen City, Guangdong Province, China; 2 Department of Nutrition and Food Hygiene, Hubei Key Laboratory of Food Nutrition and Safety, Ministry of Education Key Laboratory of Environment and Health and MOE Key Lab of Environment and Health, Key Laboratory of Environment and Health (Wuhan), Ministry of Environmental Protection, State Key Laboratory of Environment Health (Incubation), School of Public Health, Tongji Medical College, Huazhong University of Science and Technology, Wuhan

## Abstract

**Background:**

Genetic and environmental factors contribute to psoriasis, but the impact of residential environments on this condition remains uncertain. We aimed to investigate the association of residential environments with psoriasis risk and explore its interaction with genes.

**Methods:**

We retrieved data on the spatial distribution of residential environments at 300 and 1000 m buffer zones from the UK Biobank, including the proportions of natural environments, domestic gardens, green spaces, and blue spaces within these zones. We then used Cox hazard models to estimate the hazard ratios (HRs) and 95% confidence intervals (CIs) for the associations between residential environments and psoriasis risk. Lastly, we constructed polygenic risk scores to determine genetic susceptibility and further analyse the interaction with residential environments.

**Results:**

Overall, 3755 incident cases of psoriasis were documented during a median follow-up of 12.45 years. Compared with the lowest exposure quantile (Q1), Q4 exposure to natural environments (1000 m buffer: HR = 1.16, 95% CI = 1.05–1.29; 300 m buffer: HR = 1.12, 95% CI = 1.02–1.24) and green spaces (1000 m buffer: HR = 1.16, 95% CI = 1.04–1.28; 300m buffer: HR = 1.10, 95% CI = 1.00–1.21) increased the risk of psoriasis, while Q4 exposure to domestic gardens (1000 m buffer: HR = 0.85, 95% CI = 0.77–0.93; 300m buffer: HR = 0.91, 95% CI = 0.83–1.00) and Q3 exposure to blue spaces (1000 m buffer: HR = 0.89, 95% CI = 0.81–0.98) were negatively associated with psoriasis risk. Among participants with a high genetic risk, those exposed to high levels of natural environments (1000 m buffer: HR = 1.49, 95% CI = 1.15–1.93; 300 m buffer: HR = 1.39, 95% CI = 1.10–1.77) and green spaces (300 m buffer: HR = 1.30, 95% CI = 1.04–1.64) had a higher risk of psoriasis, while those exposed to blue spaces (1000 m buffer: HR = 0.78, 95% CI = 0.63–0.98) had a lower risk of psoriasis. We also observed joint effects of genetic risk and residential environments and an antagonistic additive interaction between blue spaces and genetic risk (*P* = 0.011).

**Conclusions:**

We observed that residing in natural environments and green areas increased the risk of psoriasis in our sample, while proximity to blue spaces and domestic gardens was associated to reduced risks. The association of residential environments with psoriasis risk was modified by genetic susceptibility.

Psoriasis is an inflammatory dermatosis affecting approximately 125 million people worldwide [1]. Its incidence in Europe alone ranges from 31.4 to 521.1 per 100 000 person-years, while its prevalence in adults varies from 0.27% to 11.4%, with higher rates observed among the White population [2]. Besides its direct impact, psoriasis poses a substantial burden due to its comorbidities. For example, people with this condition are up to 50% more likely to develop cardiovascular diseases [3,4]. Although the aetiology of psoriasis has not been fully explored, research has increasingly determined that genetic and environmental factors might be related to this condition.

Residential environments have long been recognised as having a major impact on human health. Green and blue spaces, for example, are critical components of our living environment; the former refers to land partially or wholly covered by grass, trees, shrubs, or vegetation, while the latter encompasses both freshwater and marine settings [5]. In fact, studies have reported that exposure to green and blue spaces positively affects pregnancy outcomes, cognitive function, mental health, diabetes, cardiovascular conditions, and premature mortality [6,7]. However, the relationship between these environmental factors and psoriasis remains largely unexplored. Previous research has indicated a positive association between air pollution and psoriasis [8]. Likewise, residential environments are closely related to an organism’s inflammatory state, with a study suggesting a negative association between neighbourhood green spaces and high-sensitivity C-reactive protein in school-aged children [9]. This is especially notable in relation to psoriasis, as its most important pathogenesis is the sustained disorder of immunity function and inflammation pathway [3]. Besides, green spaces may influence the skin’s microbiota composition, which plays an important role in immune regulation linked to psoriasis [10]. While these findings suggested a potential association between residential environments and the risk of psoriasis, direct evidence on this relationship is currently lacking.

Conversely, psoriasis is believed to be influenced by a combination of genetic and environmental factors. Genetic predisposition, for example, serves as a reliable predictor of psoriasis risk [11]. Recently, the polygenic risk score (PRS) has been used to measure the cumulative effect of multiple risk-related genetic variants to identify potentially at-risk people for psoriasis; yet the effects of genetic risk on human health are also often modified by the environment [12]. Therefore, identifying the interaction between genes and the environment could contribute to establishing more precise interventions for psoriasis.

Therefore, we sought to investigate the association between residential environments and incident risk of psoriasis by using data from the UK Biobank, including the proportion of natural environments, domestic gardens, and green and blue spaces in 1000 m and 300 m buffer zones. We also wanted to examine whether the genetic risk of psoriasis modified the associations between the residential environments and psoriasis.

## METHODS

### Study design and population

The UK Biobank is an ongoing prospective study with over half a million participants recruited from a baseline survey between 2006–2010. Its design and methods have been reported in detail previously [13]. Using the UK Biobank, we included 440 776 participants with available data on residential natural environments, domestic gardens, and green and blue spaces (Figure S1 in the **Online Supplementary Document**). After excluding participants with a history of psoriasis and missing covariates measurements, our sample comprised 385 145 participants (UK Biobank application number 88159).

### Exposure assessment

We estimated residential environments through the 2005 Generalized Land Use Database for England (GLUD) or 2007 Land Cover Map data of the Centre for Ecology and Hydrology [14–16]. We applied the GLUD to 32 482 lower-layer super-output areas (LSOAs), a geographic unit used to report small-area statistics. Each LSOA contained around 1500 residents (mean area of 4 km^2^). Area cover was accurate to approximately 10 m^2^ at the time the data were collected (2005). The GLUD divided the land cover of each LSOA into nine usage categories, including green spaces, domestic gardens, water (blue) spaces, domestic buildings, non-domestic buildings, roads, paths, railways, and others. We allocated each home location polygon an area-weighted mean of the land use percentage coverage and calculated the percentage of natural environments, domestic gardens, and green and blue spaces as a proportion of all land-use types. Drawing upon evidence linking the spatial distribution of residential environments to health outcomes and relevant public policies regarding the accessibility of green spaces, we employed proximity criteria of 300 m and 1000 m buffer zones [17]. These distances indicated a reasonable walking distance and a broader neighbourhood scale relative to the participant's home location, respectively. Other information about the measurement is available elsewhere [17].

### PRS calculation

We generated the genotype data of the UK Biobank using a custom Axiom genotyping array assaying 825 927 genetic variants, followed by genome-wide imputation. Afterwards, we constructed PRSs for psoriasis was constructed using 45 independent single-nucleotide polymorphisms (SNPs) after excluding *P* > 5 × 10^−8^ and minor allele frequency (MAF) <0.05, according to the largest genome-wide association study (GWAS) of European ancestry and previous reports [18,19] (Table S9 in the **Online Supplementary Document**). We then calculated the weighted PRS as the sum of the natural logarithmic odds ratio (ln(OR)) of each allele multiplied by the number of risk alleles (0, 1, or 2).



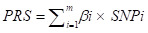



where *βi* was the per allele natural ln(OR) for SNPs and the ORs, *SNPi* was recorded as 0, 1, or 2 in terms of the number of risk alleles, and *m* was 45 (the total number of SNPs).

Another possible approach was to retrieve the psoriasis-related PRS variable directly from the UK Biobank (variable number 26269) [20]. Here we retrieved the standard PRS entirely from external GWAS data, based on which we built PRS algorithms from trait-specific meta-analyses using a Bayesian approach, where appropriate combining data across multiple ancestries and related traits. We then calculated per-individual PRS values as the genome-wide sum of the per-variant posterior effect size multiplied by allele dosage. Lastly, we standardised the PRS based on the meta-analysed summary statistics GWAS data. More details about the method of PRS construction and verification are available elsewhere [21]. In this study, we categorised PRS into low (lowest quintile (Q1)), intermediate (Q2 and Q3), and high (highest quintile (Q4)) risk.

### Diagnosing psoriasis

The main diagnostic data of psoriasis in the UK Biobank were obtained from the hospital admission records from the Hospital Episode Statistics for England, Scottish Morbidity Record Data for Scotland, and the Patient Episode Database for Wales. According to the International Classification of Diseases, 10th revision (ICD-10), we identified patients with psoriasis by the ICD codes L400, L401, L403, L404, L405, L408, and L409; ICD-9: 6960 and 6961. Other psoriasis diagnostic information includes self-reports, primary care, and death registration in the survey (UK biobank field IDs 20002, 131742, and 131743, respectively). We excluded individuals with self-reported psoriasis or psoriasis identified via hospital inpatient records at baseline. We defined follow-up time of psoriasis individuals as the difference value between the date of first recorded new onset and the baseline date, and censoring as the time of death, withdrawal from study, or the end of follow-up, whichever came first.

### Covariates

We searched the literature to identify the main relationships among exposures, outcomes, and covariates, and selected potential confounders using the directed acyclic graph (DAG) (Figure S2 in the **Online Supplementary Document**). We adjusted all analyses for the following covariates: age (continuous); sex (man or woman); ethnicity (White or non-White); body mass index (BMI) (≤25, 25–30, or ≥30 kg/m^2^); Townsend deprivation index (continuous); smoking status (never, previous, or current); alcohol intake (never, previous, or current); physical activity (poor, moderate, or vigorous); and living score (continuous). We determined an individual’s age from their dates of birth; their sex, ethnicity, smoking status, and alcohol intake from self-reported data at baseline; BMI through baseline measurements; and their area-based socioeconomic status from the postcode of residence by calculating the Townsend deprivation index. Otherwise, we defined vigorous activity as engaging in at least 150 minutes per week of moderate activity, at least 75 minutes per week of vigorous activity, or at least 150 minutes per week of mixed activity; moderate activity as engaging in 1–149 minutes per week of moderate activity, 1–74 minutes per week of vigorous activity, or 1–149 minutes per week of mixed activity; and poor activity as not engaging in any moderate or vigorous activity. Lastly, we estimated an individual’s living environment score per the indoor and outdoor living environment which contained four measures (social and private housing in poor condition, houses with central heating, air quality, and road traffic accidents).

### Statistical analysis

We first described the participants’ characteristics by their psoriasis status, presenting them as continuous variables (means and standard deviations (SDs)) or categorical variables (numbers and percentages), after which we compared them using Student’s t-test or χ^2^ test, respectively.

We then logarithmically transformed residential environments (including natural environments, domestic gardens, and green and blue space) to obtain an approximate normal distribution (continuous variables) or to categorise them into four quartiles (i.e., Q1, Q2, Q3, and Q4 for categorical variables). We also defined them as low (tertile 1), medium (tertile 2), and high (tertile 3) exposure levels to explore the joint effects. We then used multivariable Cox proportional hazards models to estimate hazard ratios (HRs) and 95% confidence intervals (CIs) for the associations between residential environment space and incident psoriasis risk. We evaluated the proportional hazards assumption by Schoenfeld residuals. We then performed the linear trend test across ordinal exposure groups in models using special integer values (1, 2, 3, and 4) and assessed the dose-response relationships between environment space and psoriasis by restricted cubic spline regression, with 3 knots placed at the 25th, 50th, and 75th percentile. We used the population-attributable risk (PAR) to estimate the proportion of psoriasis attributable to environment space, using the prevalence and adjusted HR of each category.

We examined the association of the interaction between genetic risk and environment space with the incident psoriasis using Cox proportional hazard regression models after further adjusting the genotyping batch and genetic principal components and only including White individuals. We evaluated the additive interaction by the relative excess risk because of the interaction and the attributable proportion due to interaction, whereby the 95% CI containing 0 indicated no additive interaction. We tested for multiplicative interaction by adding product terms in the Cox models.

To identify susceptible subgroups, we conducted stratiﬁed analyses by age (≤60 or >60 years), sex (man or woman), BMI (≤25, 25–30, or ≥30 kg/m^2^), physical activity (poor, moderate, vigorous), Townsend deprivation index (≤ median or > median), and living score (≤ median or > median). We also performed several sensitivity analyses by further adjusting for HbA1c, triglyceride, and systolic blood pressure; further adjusting for the levels of lymphocyte percentage, white blood cell count, and C-reactive protein; further adjusting for cancer, diabetes, and cardiovascular disease; by restricting the analysis to participants living at the same address >3 years; further adjusting by the multiple imputation of covariates.

We performed all analyses in SAS, version 9.4 (SAS, Cary, North Carolina, USA) or R, version 4.2.2 (R Core Team, Vienna, Austria).

## RESULTS

### Baseline characteristics

The mean age of study participants was 56.5 years, while 54.3% were female (**Table 1**). During a median follow-up for 12.45 years (5 242 267 person-years), 3755 incident cases of psoriasis were documented among 385 145 total participants, with the incidence density being 71.63 per 100 000 person-years. Psoriasis patients were more likely to be older, male, white, smokers, alcohol drinkers, obese, have a higher Townsend deprivation index, and have low physical activity compared to non-psoriasis participants. The residential environments of patients with psoriasis had had a lower proportion of domestic gardens and blue spaces, and a higher proportion of natural environments and green spaces. Per the Pearson correlation matrix, natural environments had a strong, positive correlation with green spaces, and a poor correlation with blue spaces. Domestic gardens were negatively correlated with other environments (Table S1 and Figure S3 in the **Online Supplementary Document**).

**Table 1:** Basic characteristics between the psoriasis and non-psoriasis participants*

	Total (n = 385 145)	Non-psoriasis (n = 381 390)	Psoriasis (n = 3755)	*P-*value
**Age in years, x̄ (SD)**	56.5 (8.1)	56.5 (8.1)	57.6 (7.8)	<0.001†
**Sex**				<0.001‡
Female	209 083 (54.3)	207 187 (54.3)	1896 (50.5)	
Male	176 062 (45.7)	174 203 (45.7)	1859 (49.5)	
**Race/ethnicity**				<0.001‡
White	364 588 (94.7)	360 983 (94.6)	3605 (96.0)	
Non-White	20 557 (5.3)	20 407 (5.4)	150 (4.0)	
**BMI in kg/m^2^**				<0.001‡
≤25	129 731 (33.7)	1287 20 (33.7)	1011 (26.9)	
25-30	164 056 (42.6)	162 449 (42.6)	1607 (42.8)	
≥30	91 358 (23.7)	90 221 (23.7)	1137 (30.3)	
**Alcohol intake**				0.022‡
Never	15 949 (4.1)	15 809 (4.1)	140 (3.7)	
Ever	13 254 (3.4)	13 097 (3.4)	157 (4.2)	
Current	355 942 (92.5)	352 484 (92.5)	3458 (92.1)	
**Smoking status**				<0.001‡
Never	211 851 (55.0)	210 235 (55.1)	1616 (43.0)	
Ever	134 924 (35.0)	133 378 (35.0)	1546 (41.2)	
Current	38 370 (10.0)	37 777 (9.9)	593 (15.8)	
**Physical activity**				<0.001‡
Poor	66 396 (17.2)	65 610 (17.2)	786 (20.9)	
Moderate	190 042 (49.4)	188 296 (49.4)	1746 (46.5)	
Vigorous	128 707 (33.4)	127 484 (33.4)	1223 (32.6)	
**Townsend deprivation index, x̄ (SD)**	−1.4 (3.0)	−1.4 (3.0)	−1.1 (3.1)	<0.001†
**Living score, x̄ (SD)**	18.5 (15.1)	18.5 (15.1)	19.1 (15.1)	0.011†

BMI – body mass index, SD – standard deviation, x̄ – mean

*Presented as n (%) unless specified otherwise.

†Student’s *t*-test.

‡χ^2^ test.

### Residential environment space and psoriasis risk

The highest exposure quantile (Q4) of natural environments (1000 m buffer: HR = 1.16, 95% CI = 1.05–1.29; 300 m buffer: HR = 1.12, 95% CI = 1.02–1.24) and green spaces (1000 m buffer: HR = 1.16, 95% CI = 1.04–1.28; 300 m buffer: HR = 1.10, 95% CI = 1.00–1.21) increased the incident risk of psoriasis (*P*-value for trend <0.05). Compared with the lowest exposure (Q1), higher exposure to domestic gardens for Q4 (1000 m buffer: HR = 0.85, 95% CI = 0.77–0.93; 300 m buffer: HR = 0.91, 95% CI = 0.83–1.00) and blue spaces for Q3 (1000 m buffer: HR = 0.89, 95% CI = 0.81–0.98) were negatively associated with psoriasis risk (*P*-value for trend <0.05). These associations were also linearly presented in environment space and psoriasis risk (**Table 2**; Figure S4 in the **Online Supplementary Document**). The PAR% for psoriasis caused by the high exposure to natural environments were 4.72% and 3.29% in 1000 m and 300 m buffer zones, respectively, while those for green spaces were 3.53% and 3.72% in 1000 m and 300 m buffer zones, respectively. The PAR% of psoriasis due to low exposure to domestic gardens were 3.44% and 3.53% in 1000 m and 300 m buffer zones, respectively, while the PAR% for blue spaces were 2.57% and 3.04%, in the 1000m buffer and 300m buffer areas, respectively (Table S2 in the **Online Supplementary Document**). The results did not differ substantially in the sensitivity analyses (Table S3–7 in the **Online Supplementary Document**).

**Table 2:** Associations of residential natural environment, domestic garden, green space, and blue space with the risk of incident psoriasis, presented as HRs (95% CIs)*

	Continuous	*P*-value	Q1	Q2	*P*-value	Q3	*P*-value	Q4	*P*-value	*P*-value for trend
**Natural environment**										
1000 m buffer	1.06 (1.01–1.11)	0.029	ref	1.11 (1.01–1.23)	0.027	1.10 (0.99–1.21)	0.085	1.16 (1.05–1.29)	0.005	0.014
300 m buffer	1.03 (1.01–1.06)	0.013	ref	1.05 (0.95–1.15)	0.341	1.06 (0.97–1.17)	0.214	1.12 (1.02–1.24)	0.019	0.020
**Domestic garden**										
1000 m buffer	0.93 (0.89–0.98)	0.008	ref	0.93 (0.85–1.02)	0.125	0.99 (0.91–1.08)	0.819	0.85 (0.77–0.93)	<0.001	0.004
300 m buffer	0.94 (0.90–0.99)	0.012	ref	1.00 (0.91–1.09)	0.935	0.98 (0.89–1.07)	0.585	0.91 (0.83–1.00)	0.043	0.042
**Green space**										
1000 m buffer	1.08 (1.00–1.16)	0.049	ref	1.03 (0.93–1.13)	0.575	1.08 (0.98–1.20)	0.122	1.16 (1.04–1.28)	0.006	0.003
300 m buffer	1.04 (0.99–1.09)	0.114	ref	0.98 (0.89–1.07)	0.589	1.03 (0.94–1.13)	0.576	1.10 (1.00–1.21)	0.062	0.035
**Blue space**										
1000 m buffer	0.97 (0.91–1.02)	0.230	ref	0.98 (0.90–1.07)	0.652	0.89 (0.81–0.98)	0.012	0.93 (0.85–1.02)	0.120	0.033
300 m buffer	0.96 (0.91–1.02)	0.226	ref	0.96 (0.88–1.05)	0.416	0.94 (0.86–1.03)	0.184	0.93 (0.85–1.02)	0.123	0.104

CI – confidence interval, Q – quantile

*Models were adjusted for age, sex, body mass index, ethnicity, Townsend deprivation index, smoking status, alcohol intake, physical activity, and living score.

In the stratified analyses (Figure S5 in the **Online Supplementary Document**), the associations of psoriasis with natural environments, green spaces, and domestic gardens seemed stronger in men and young people, while the association with blue spaces was stronger in women and old people. We also saw stronger associations among participants living in areas with a lower deprivation index and living environment score.

**Figure 1:**
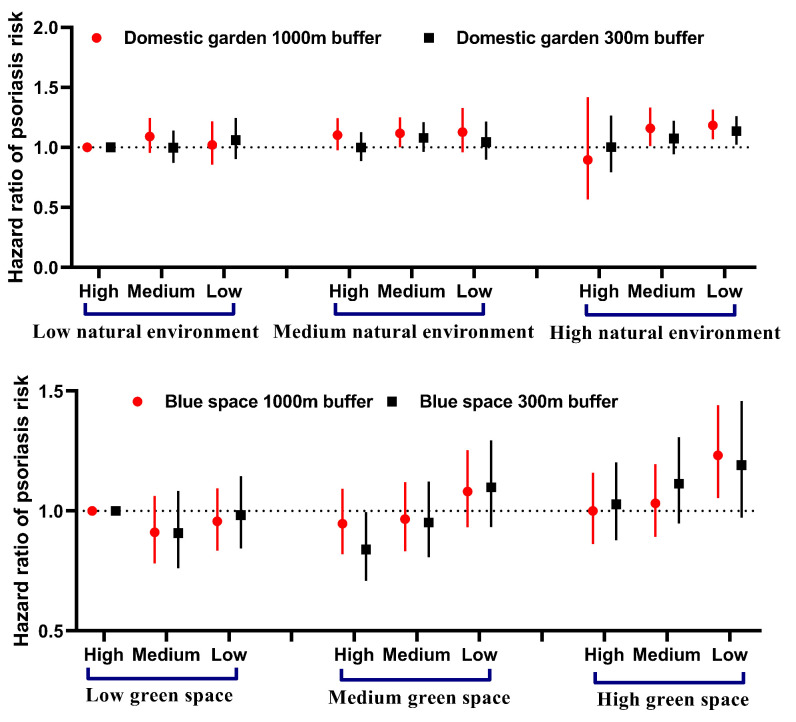
Joint effect of residential natural environment and domestic garden, green space and blue space on the risk of incident psoriasis. Models were adjusted for age, sex, body mass index, ethnicity, Townsend deprivation index, smoking status, alcohol intake, physical activity, and living score. Low exposure: tertile 1. Medium exposure: tertile 2. High exposure: tertile 3.

We further analysed the joint effects among each environment space. Compared with participants living with low exposure to natural environments and high exposure to domestic gardens, those with high exposure to natural environments but low exposure to domestic gardens had a higher risk of incident psoriasis (1000 m buffer: HR = 1.18, 95% CI = 1.07–1.32; 300 m buffer: HR = 1.14, 95% CI = 1.02–1.26), while participants living with high exposure to green spaces and low exposure to blue spaces had a higher risk of psoriasis (1000 m buffer: HR = 1.23, 95% CI = 1.05–1.44; 300 m buffer: HR = 1.19, 95% CI = 0.97–1.46) **(****Figure 1**). We also observed the antagonistic additive interactions between green space and blue space (*P* = 0.019) (Table S8 in the **Online Supplementary Document**).

### Residential environment space and genetic risk

**Figure 2:**
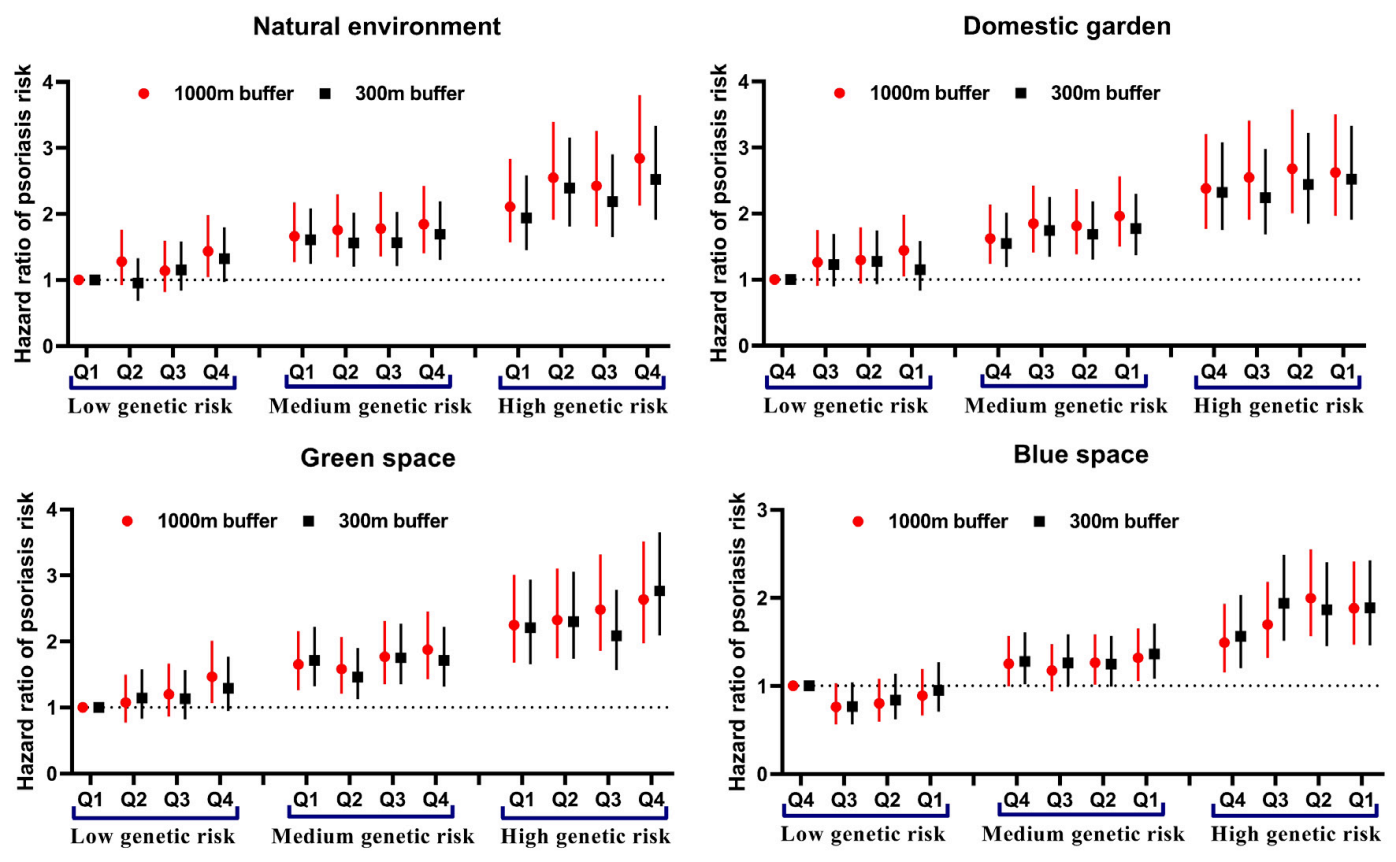
HRs of psoriasis risk based on residential natural environment, domestic garden, green space and blue space stratified by PRS. Models were adjusted for age, sex, body mass index, ethnicity, smoking status, alcohol intake, Townsend deprivation index, physical activity, living score, genotyping batch, and genetic principal components. **Panel A. **1000 m buffer. **Panel B.** 300 m buffer.

**Figure 3:**
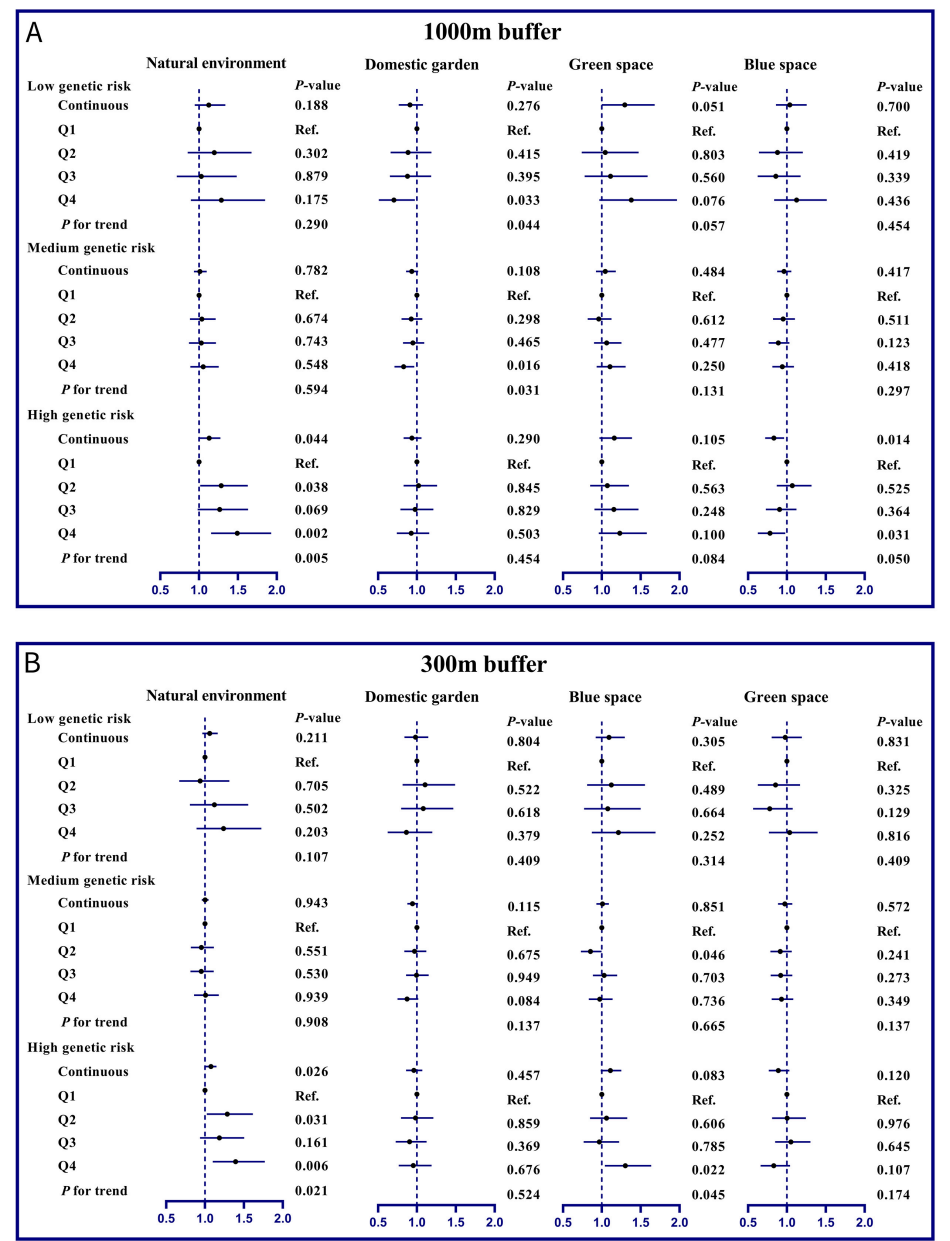
Joint effects of residential natural environment, domestic garden, green space and blue space with PRS on the risk of psoriasis risk. Models were adjusted for age, sex, body mass index, ethnicity, smoking status, alcohol intake, Townsend deprivation index, physical activity, living score, genotyping batch, and genetic principal components. PRS – polygenic risk score, Q – quantile, RA – rheumatoid arthritis.

We constructed psoriasis-related PRS and observed an increase in the risk of incident psoriasis across genetic risk categories (*P*-value for trend <0.001) (Tables S9, S10, and S12 in the **Online Supplementary Document**). Among participants with a high genetic risk, those exposed to Q4 levels of natural environments (1000 m buffer: HR = 1.49, 95% CI = 1.15–1.93; 300 m buffer: HR = 1.39, 95% CI = 1.10–1.77) or green spaces (300 m buffer: HR = 1.30, 95% CI = 1.04–1.64) had a higher risk of psoriasis compared to those exposed to Q1 levels. Conversely, participants with Q4 exposure to blue spaces had a lower risk of psoriasis (1000 m buffer: HR = 0.78, 95% CI = 0.63–0.98) (**Figure 2**). We saw similar findings from the analysis stratified by standard PRS (Figure S6 in the **Online Supplementary Document**). We next estimated the joint effect of environment space and PRS on the risk of incident psoriasis. Here, participants with high genetic risk and Q4 exposure to natural environments (1000 m buffer: HR = 2.84, 95% CI = 2.13–3.80; 300 m buffer: HR = 2.53, 95% CI = 1.91–3.34) or green spaces (1000 m buffer: HR = 2.63, 95% CI = 1.97–3.52; 300 m buffer: HR = 2.76, 95% CI = 2.09–3.65) had a higher risk of incident psoriasis compared to those with a low PRS plus Q1 (**Figure 3**). The joint effects of PRS with domestic gardens and blue spaces were relatively poor. Furthermore, we observed an antagonistic additive interaction between blue space and PRS (*P* = 0.011), while we saw no significant interaction between genetic risk and other environmental space (Table S11 in the **Online Supplementary Document**). The results were consistent when analysed per the standard PRS from the UK Biobank (Figure S7 and Table S13 in the **Online Supplementary Document**).

## DISCUSSION

This is the first study to investigate the effects of residential environments on the incident risk of psoriasis in a large prospective cohort. We observed significant associations of residential natural environments and green spaces with an increased psoriasis risk. Inversely, domestic gardens and blue spaces were negatively associated with the risk of psoriasis. Moreover, individuals with a high genetic predisposition who were simultaneously exposed to a high percentage of natural environments and green spaces had the greatest relative increase in psoriasis risk. We otherwise found no significant interaction between genetic risk and natural environments and green spaces, yet observed an antagonistic additive interaction with blue spaces.

We investigated the association between residential environments and psoriasis risk on both the scale of reasonable walking distance (300 m buffer zone) and that of a large neighbourhood (1000 m buffer zone). Our findings provide epidemiological evidence that exposure to natural environments and green spaces increases the incidence of psoriasis. However, this contradicts most previous reports suggesting a positive effects of green spaces on human health [22–24]. Psoriasis is known for its impairing effect of the skin’s barrier function [25]; an analysis of skin samples from both psoriasis patients and controls using 16S rRNA showed notable discrepancies in microbiota between the two groups, highlighting pathogenically relevant cutaneous microbial changes in the patients with psoriasis [10,26]. Microorganisms can be passed between humans and their surroundings to mediate the effects of the environment [27]. A survey conducted across diverse urban green space environments in three cities revealed that exposure to green space altered skin and nasal microbial diversity [28]. The above studies [25–28] suggests that the skin microbiome plays a role in the association between green spaces and psoriasis. Additionally, certain studies showed that exposure to green spaces may increase the risk of allergic diseases in children, such as asthma and allergic rhinitis [29], suggesting that they may be involved as a cause in immune-related disorders, especially given that psoriasis is a condition strongly linked with immunity. On whole, these findings suggest that exposure to natural vegetation could potentially increase susceptibility to psoriasis.

In contrast, we found that domestic gardens and blue spaces have a protective role against psoriasis. Domestic gardens are usually considered part of the built environment, which generally has lower exposure to bacteria and microbes compared to a natural one [30]. Our analyses of joint effects also showed that individuals exposed to a combination of more natural environments and fewer domestic gardens had the greatest risk of psoriasis. Previous research reported that people were less stressed and had higher life satisfaction in blue spaces than in other settings [31]. A relatively moist environment is notably good for psoriasis, which manifests through the dry skin tissue. Moreover, green and blue spaces may have distinct pollen profiles, potentially leading to a variety of different antigens [32]. Hence, they may play different roles in human health. Interestingly, we found that blue spaces have an antagonistic additive interaction with green spaces on psoriasis risk, highlighting the importance of exposure to the former. While the overall role the environment plays in the development of psoriasis not well understood and the relevant factors have not been identified, our study provides some insights that psoriasis cases would be reduced by 2.57–4.72% among the total population through the reduction of natural environments and green spaces or the increase of domestic gardens and blue spaces.

In our analysis, residential environments played different roles in different subgroups. We found that young people and men had a higher psoriasis risk when exposed to the natural environment and green space, but a lower risk when exposed to domestic garden. This might be due to these groups engaging in more outdoor activities and therefore being more affected by surroundings. Consistently, green spaces and domestic gardens were associated with a higher and lower risk of psoriasis, respectively, among people with more physical activity. Notably, the associations between residential environments and psoriasis were stronger among participants living in areas with a lower Townsend deprivation index. This is partly attributed to the fact that more developed areas tend to have higher levels of air and noise pollution. Moreover, the urban residents may be at a higher risk for mental and psychiatric disorders [33], thus reinforcing the influence of environmental factors on psoriasis.

Genetic factors have been considered the largest contributor to the incidence of psoriasis. In our study, both our constructed PRS and standard PRS calculated from the UK Biobank have shown to be robust predictors of psoriasis risk, meaning that the higher the genetic risk in the population, the higher the risk of psoriasis being increased by exposure to natural environments. Furthermore, we observed joint effects between residential environments and genetic factors on psoriasis risk, implying that people with high genetic susceptibility to psoriasis should be attentive of the spatial distribution of land within their living environment, despite there being no interaction of genetic risk with residential natural environments, green spaces, and domestic gardens. Our results showed that 12% of psoriasis risk could be attributed to additive interactions between genetic risk and exposure to blue spaces at a 1000 m buffer zone, suggesting that blue spaces may contribute to reducing the incidence of psoriasis in populations with high genetic susceptibility. A wide range of mechanisms has been proposed to explain why genetic and environmental factors are associated with psoriasis risk. For example, psoriasis-related genetic variants may affect individuals’ immune response, oxidative damage, and anti-inflammatory effects [34]. Green spaces and water spaces may also be involved in psoriasis by affecting immune response and inflammatory pathways [35–37].

This study has several limitations. First, the data on residential environments and most covariates were available only at baseline, preventing us from investigating changes in residential environments. Second, although we considered some factors, future research should comprehensively estimate the role of lifestyle in the association between residential environments and psoriasis. Third, there are still additional environmental factors such as radio-frequent radiation and climate change during the follow-up period that may have biased our analyses. Fourth, the distribution of blue spaces is not dramatically different and relatively small because the UK is a well-industrialised country. Fifth, we only estimated the exposure of the residential environment at 300 m and 1000 m buffer zones. A more accurate exposure for each individual should be assessed in the future. Sixth, hospital inpatient records may have missed people with less severe clinical symptoms and late-onset psoriasis cases. Seventh, the relatively homogenous sample of ethnicities in the UK Biobank limits the generalisability of our findings to other populations. Eighth, while this study offers several mechanistic speculations, further investigations should explore the underlying mechanisms in greater depth. Finally, our study had different findings than prior research, and its observational prevents us from establishing direct causality. Future studies should clarify the relationship between residential environments and psoriasis via other methodologies, such as animal studies, Mendelian randomisation, or randomised clinical trials.

## CONCLUSIONS

High exposure to residential natural environments and green spaces was a notable risk factor for psoriasis, while exposure to blue spaces and domestic gardens was associated with lower risks of psoriasis. The associations between environment spaces and the risk of incident psoriasis were strengthened by genetic susceptibility.

## Additional material


Online Supplementary Document
