# Supplementary material for: Spatial distribution of residential environment, genetic susceptibility, and psoriasis: A prospective cohort study
Source: J Glob Health. 2024 Aug 6;14:04139. doi: 10.7189/jogh.14.04139 (PMC11301618; doi:10.7189/jogh.14.04139)
Supplement: Online Supplementary Document [file jogh-14-04139-s001.pdf]

Supplementary materials

Figures:

Figure S1. The flowchart of participant selection from UK Biobank.

Figure S2. Directed acyclic graph.

Figure S3. Pearson's correlation matrix among residential natural environment, domestic garden, green space, and blue space.

Figure S4. The dose-response relationship of residential natural environment, domestic garden, green space, and blue space with incident psoriasis assessed by restricted cubic spline regression.

Figure S5. Stratified analyses of the associations of residential natural environment, domestic garden, green space, and blue space with incident psoriasis.

Figure S6. Hazard ratio of psoriasis risk based on residential natural environment, domestic garden, green space and blue space stratified by Standard PRS.

Figure S7. Joint effects of residential natural environment, domestic garden, green space and blue space with Standard PRS on the risk of psoriasis risk.

Tables:

Table S1. Distribution of residential natural environment, domestic garden, green space, and blue space at baseline.

Table S2. PAR% of residential natural environment, domestic garden, green space, and blue space with the risk of incident psoriasis.

Table S3. Associations of residential natural environment, domestic garden, green space, and blue space with the risk of incident psoriasis after further adjusting for HbA_{1c}, triglyceride and systolic blood pressure.

Table S4. Associations of residential natural environment, domestic garden, green space, and blue space with the risk of incident psoriasis after further adjusting for lymphocyte percentage, white blood cell count, and C-reactive protein.

Table S5. Associations of residential natural environment, domestic garden, green space, and blue space with the risk of incident psoriasis after further adjusting for cancer, diabetes, and cardiovascular disease.

Table S6. Associations of residential natural environment, domestic garden, green space, and blue space with the risk of incident psoriasis after restricting participants living at the same address >3 years.

Table S7. Associations of residential natural environment, domestic garden, green space, and blue space with the risk of incident psoriasis with the imputation of covariates.

Table S8. Additive and multiplicative interactions of residential natural environment and domestic garden, and green space and blue space on the risk of incident psoriasis.

Table S9. Summary results of SNPs used for PRS in the study.

Table S10. Hazard ratio of psoriasis risk based on PRS.

Table S11. Additive and multiplicative interactions of residential natural environment, domestic garden, green space, and blue space with PRS on the risk of incident psoriasis.

Table S12. Hazard ratio of psoriasis risk based on Standard PRS.

Table S13. Additive and multiplicative interactions of residential natural environment, domestic garden, green space, and blue space with Standard PRS on the risk of incident psoriasis.

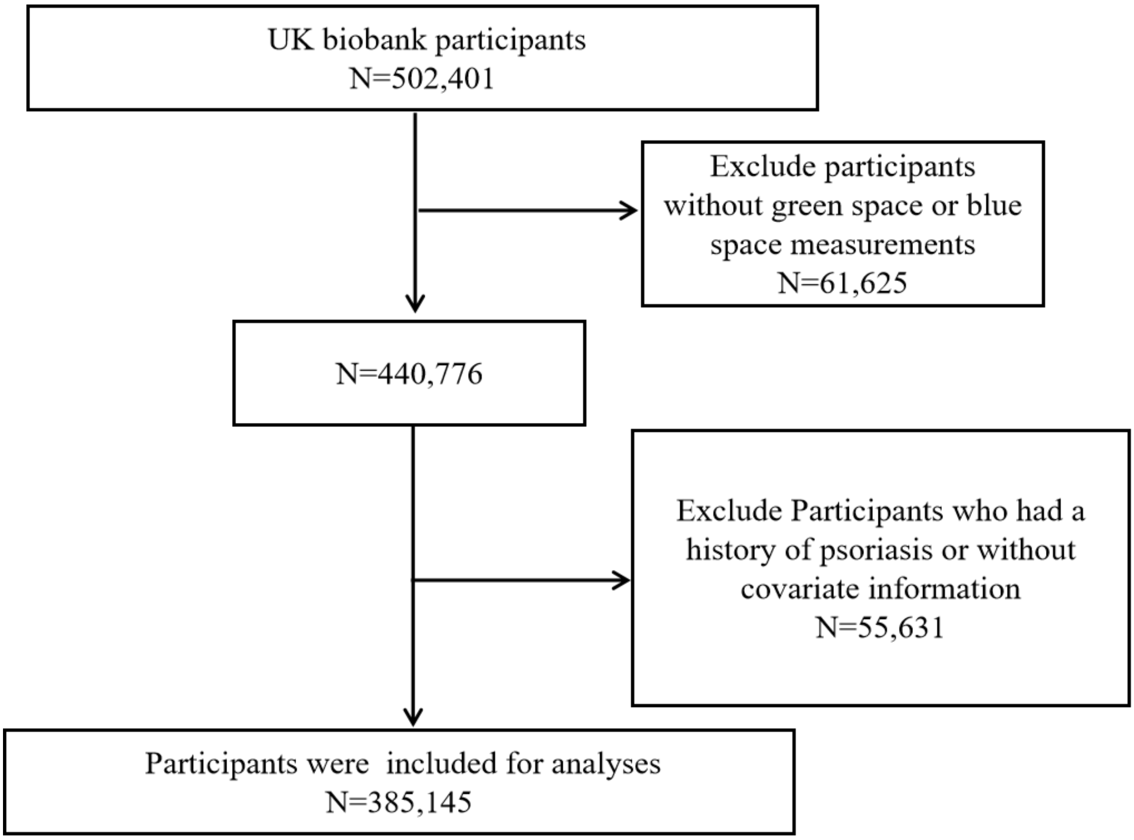

Figure S1. The flowchart of participant selection from UK Biobank.

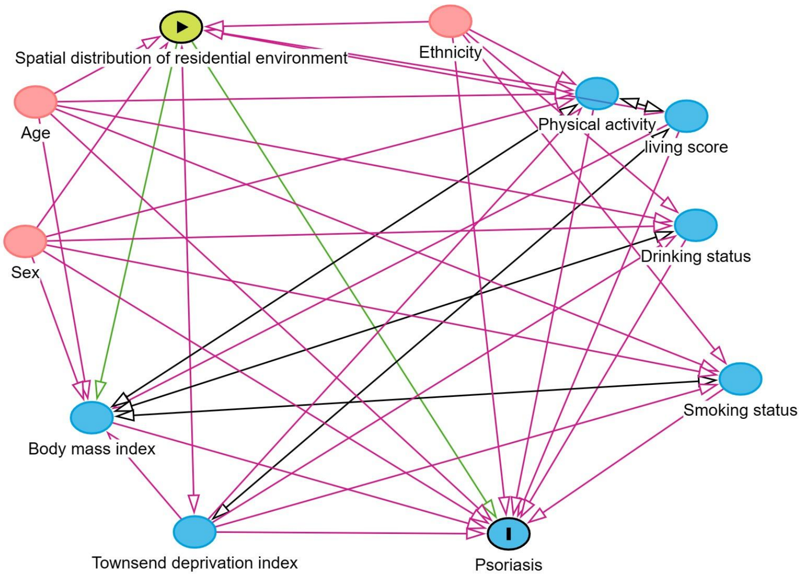

Figure S2. The directed acyclic graphs for the effect of spatial distribution of residential environment on psoriasis.

Table S1. Distribution of residential natural environment, domestic garden, green space, and blue space at baseline.

Variables	Total (N=385145)	Non-psoriasis (N=381390)	Psoriasis (N=3755)
Natural environment, 1000m buffer (%)			
Mean ± standard deviation	41.4 ± 25.8	41.4 ± 25.8	41.5 ± 25.5
Min–Max	0.0–100.0	0.0–100.0	0.0–100.0
Median (Inter-quartile Range)	37.5 (19.7–59.8)	37.5 (19.7–59.7)	37.6 (20.3–59.9)
Natural environment, 300m buffer (%)			
Mean ± standard deviation	26.8 ± 25.5	26.8 ± 25.5	27.1 ± 25.4
Min–Max	0.0–100.0	0.0–100.0	0.0–100.0
Median (Inter-quartile Range)	19.6 (6.3–40.6)	19.6 (6.3–40.6)	20.1 (6.7–41.3)
Domestic garden, 1000m buffer (%)			
Mean ± standard deviation	24.4 ± 11.3	24.4 ± 11.3	23.7 ± 11.0
Min–Max	0.1–66.1	0.1–66.1	0.2–58.9
Median (Inter-quartile Range)	24.5 (16.6–32.2)	24.5 (16.6–32.2)	23.8 (15.9–30.9)
Domestic garden, 300m buffer (%)			
Mean ± standard deviation	31.4 ± 14.7	31.4 ± 14.7	30.5 ± 14.6
Min–Max	0.0–77.4	0.0–77.4	0.2–72.2
Median (Inter-quartile Range)	32.1 (21.0–42.0)	32.1 (21.0–42.1)	31.2 (20.3–40.7)
Green space, 1000m buffer (%)			
Mean ± standard deviation	45.3 ± 21.7	45.3 ± 21.7	45.6 ± 21.7
Min–Max	4.4–99.2	4.4–99.2	7.4–98.6
Median (Inter-quartile Range)	41.9 (27.6–60.4)	41.9 (27.6–60.4)	42.9 (28.1–61.2)
Green space, 300m buffer (%)			
Mean ± standard deviation	35.5 ± 23.4	35.5 ± 23.4	36.0 ± 23.5
Min–Max	0.2–99.2	0.2–99.2	0.5–98.6
Median (Inter-quartile Range)	29.7 (17.3–48.8)	29.7 (17.3–48.8)	30.4 (17.6–49.7)
Blue space, 1000m buffer (%)			
Mean ± standard deviation	1.3 ± 2.5	1.3 ± 2.5	1.2 ± 2.3
Min–Max	0.0–59.4	0.0–59.4	0.0–43.3
Median (Inter-quartile Range)	0.5 (0.2–1.3)	0.5 (0.2–1.3)	0.5 (0.2–1.3)
Blue space, 300m buffer (%)			
Mean ± standard deviation	0.9 ± 2.9	0.9 ± 2.9	0.8 ± 2.6
Min–Max	0.0–97.9	0.0–97.9	0.0–64.3
Median (Inter-quartile Range)	0.1 (0.0–0.6)	0.1 (0.0–0.6)	0.1 (0.0–0.6)

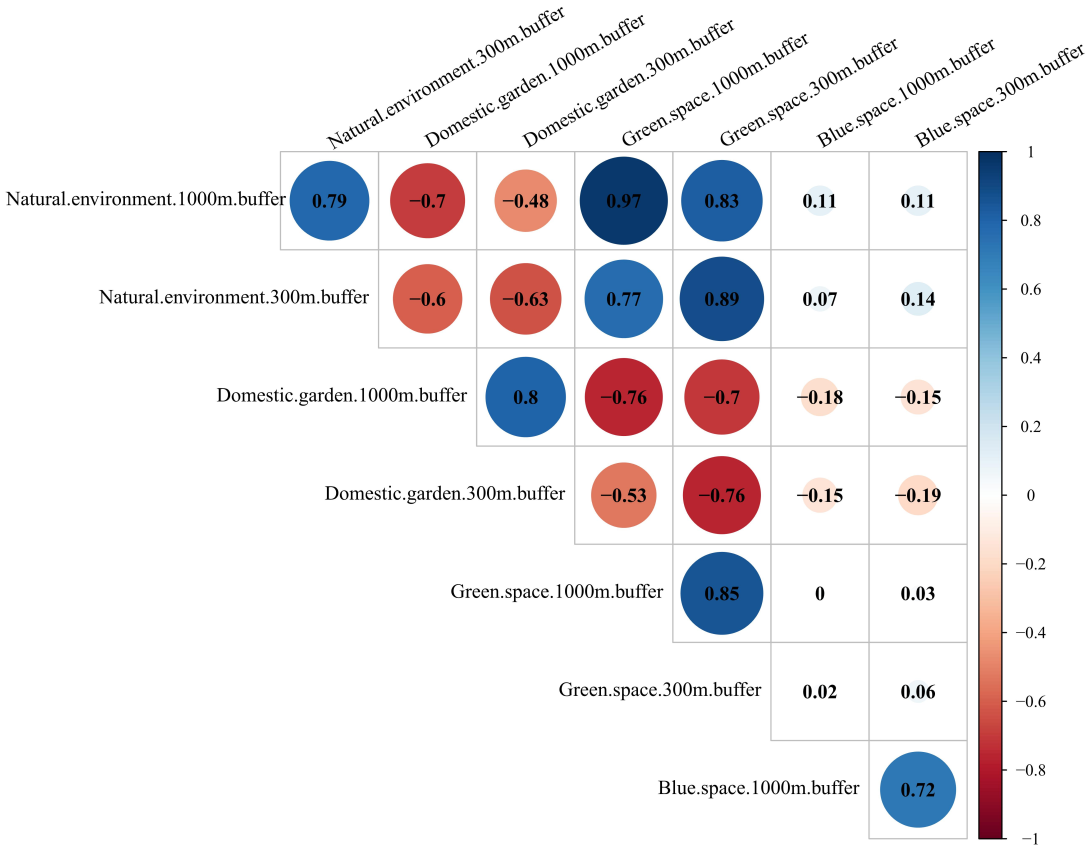

Figure S3. Pearson’s correlation matrix among residential natural environment, domestic garden, green space, and blue space.

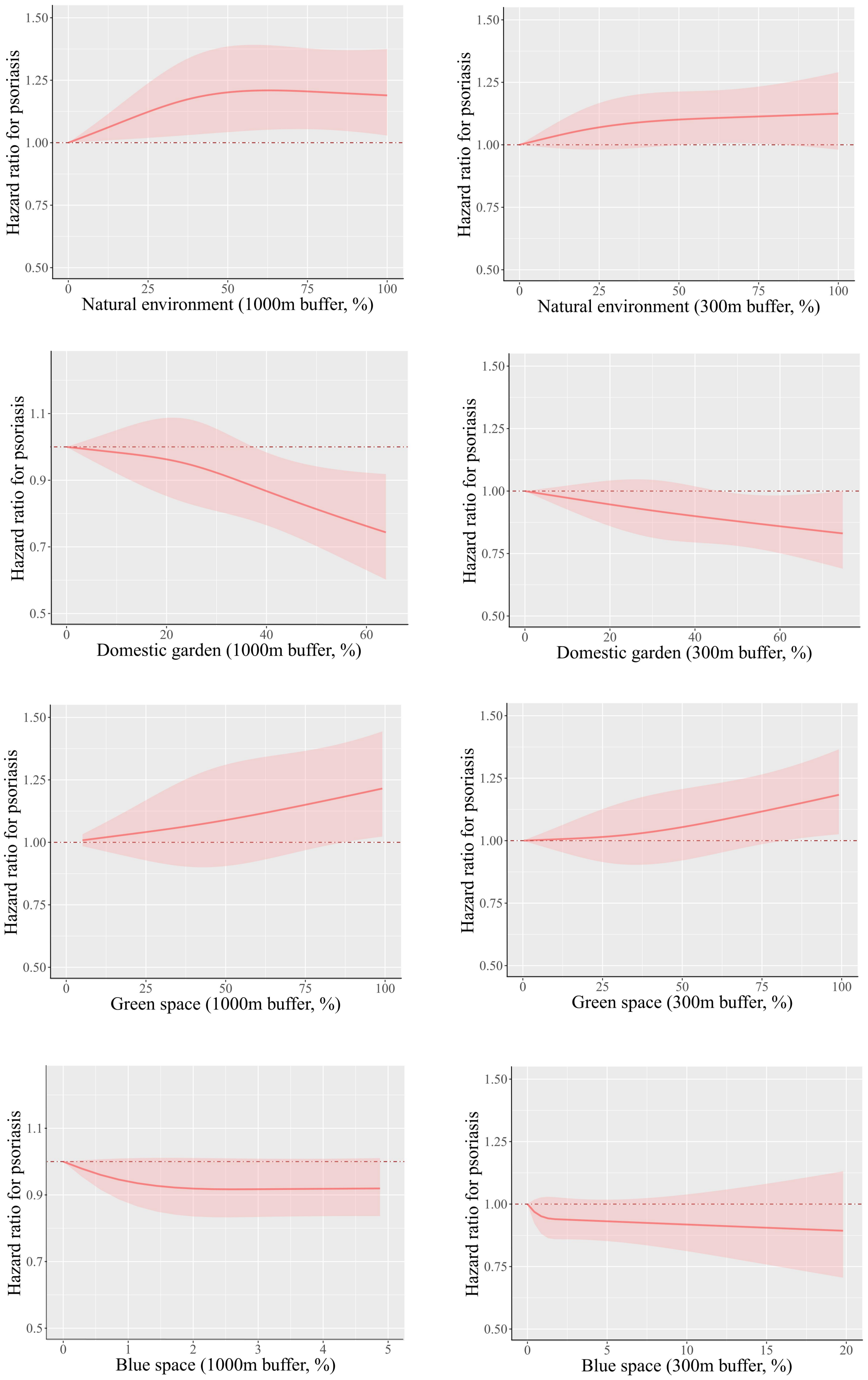

Figure S4. The dose-response relationship of residential natural environment, domestic garden, green space, and blue space with incident psoriasis assessed by restricted cubic spline regression. Models were adjusted for age, sex, body mass index, ethnicity, Townsend deprivation index, smoking status, alcohol intake, physical activity, and living score.

Table S2. PAR% of residential natural environment, domestic garden, green space, and blue space with incident psoriasis.

	PAR% (95% CI)		
	Low exposure	Medium exposure	High exposure
Natural environment			
1000m buffer	Ref.	2.79 (0.00, 5.72)	4.72 (1.59, 7.87)
300m buffer	Ref.	1.08 (-1.58, 3.84)	3.29 (0.46, 6.19)
Domestic garden			
1000m buffer	3.44 (0.75, 6.16)	2.63 (0.00, 5.37)	Ref.
300m buffer	3.53 (0.82, 6.28)	1.81 (-0.83, 4.54)	Ref.
Green space			
1000m buffer	Ref.	0.98 (-1.78, 3.81)	3.53 (0.53, 6.62)
300m buffer	Ref.	-0.33 (2.96, 2.38)	3.72 (0.92, 6.57)
Blue space			
1000m buffer	2.57 (0.00, 5.22)	-0.26 (-2.85, 2.38)	Ref.
300m buffer	3.04 (0.36, 5.75)	1.40 (-1.20, 4.11)	Ref.

Models were adjusted for age, sex, body mass index, ethnicity, Townsend deprivation index, smoking status, alcohol intake, physical activity, and living score.

Low exposure: tertile 1; Medium exposure: tertile 2; High exposure: tertile 3.

Abbreviations: PAR, Population attributable risk; CI, confidence interval.

Table S3. Associations of residential natural environment, domestic garden, green space, and blue space with the risk of incident psoriasis after further adjusting for HbA_{1c}, triglyceride and systolic blood pressure.

	Hazard ratio (95% CI)					*P* for trend
	Continuous	Q1	Q2	Q3	Q4	
Case/total			3179/332113			
Natural environment						
1000m buffer	1.06 (1.01, 1.12)	Ref.	1.13 (1.02, 1.25)	1.09 (0.97, 1.22)	1.18 (1.06, 1.33)	0.014
P-value	0.031		0.022	0.137	0.004	
300m buffer	1.03 (1.00, 1.06)	Ref.	1.05 (0.95, 1.17)	1.06 (0.96, 1.18)	1.14 (1.02, 1.26)	0.020
P-value	0.026		0.323	0.245	0.017	
Domestic garden						
1000m buffer	0.95 (0.89, 1.00)	Ref.	0.93 (0.84, 1.02)	0.99 (0.90, 1.09)	0.84 (0.76, 0.93)	0.006
P-value	0.046		0.113	0.829	0.001	
300m buffer	0.95 (0.91, 1.00)	Ref.	1.02 (0.93, 1.13)	0.99 (0.90, 1.10)	0.91 (0.82, 1.01)	0.070
P-value	0.061		0.634	0.911	0.078	
Green space						
1000m buffer	1.07 (0.99, 1.16)	Ref.	1.01 (0.91, 1.12)	1.06 (0.95, 1.18)	1.14 (1.02, 1.28)	0.010
P-value	0.093		0.898	0.292	0.018	
300m buffer	1.03 (0.98, 1.09)	Ref.	0.99 (0.90, 1.09)	1.02 (0.92, 1.13)	1.10 (0.99, 1.22)	0.062
P-value	0.254		0.835	0.746	0.074	
Blue space						
1000m buffer	0.96 (0.90, 1.02)	Ref.	0.99 (0.89, 1.09)	0.89 (0.81, 0.98)	0.92 (0.83, 1.01)	0.026
P-value	0.209		0.768	0.021	0.093	
300m buffer	0.97 (0.91, 1.03)	Ref.	0.96 (0.87, 1.06)	0.96 (0.87, 1.06)	0.93 (0.84, 1.03)	0.162
P-value	0.333		0.398	0.385	0.142	

Models were adjusted for age, sex, body mass index, ethnicity, Townsend deprivation index, smoking status, alcohol intake, physical activity, living score, HbA_{1c}, triglyceride and systolic blood pressure..

Abbreviations: CI, confidence interval; Q, quantile; HbA_{1c}: Glycated hemoglobin.

Table S4. Associations of residential natural environment, domestic garden, green space, and blue space with the risk of incident psoriasis after further adjusting for lymphocyte percentage, white blood cell count, and C-reactive protein.

	Hazard ratio (95% CI)					*P* for trend
	Continuous	Q1	Q2	Q3	Q4	
Case/total			3388/350555			
Natural environment						
1000m buffer	1.05 (1.00, 1.11)	Ref.	1.10 (1.00, 1.22)	1.08 (0.97, 1.20)	1.16 (1.04, 1.30)	0.022
P-value	0.046		0.057	0.17	0.009	
300m buffer	1.03 (1.01, 1.06)	Ref.	1.05 (0.95, 1.16)	1.06 (0.96, 1.18)	1.12 (1.02, 1.25)	0.030
P-value	0.018		0.314	0.239	0.025	
Domestic garden						
1000m buffer	0.94 (0.89, 0.99)	Ref.	0.94 (0.86, 1.03)	0.99 (0.91, 1.09)	0.84 (0.76, 0.92)	0.004
P-value	0.026		0.18	0.893	<0.001	
300m buffer	0.95 (0.91, 1.00)	Ref.	1.00 (0.91, 1.10)	1.00 (0.91, 1.10)	0.90 (0.82, 1.00)	0.062
P-value	0.038		0.935	0.992	0.046	
Green space						
1000m buffer	1.07 (0.99, 1.16)	Ref.	1.02 (0.92, 1.13)	1.07 (0.96, 1.19)	1.15 (1.03, 1.28)	0.013
P-value	0.099		0.718	0.226	0.013	
300m buffer	1.03 (0.98, 1.09)	Ref.	0.99 (0.89, 1.09)	1.03 (0.93, 1.14)	1.09 (0.99, 1.21)	0.127
P-value	0.203		0.771	0.562	0.082	
Blue space						
1000m buffer	0.96 (0.91, 1.02)	Ref.	0.97 (0.88, 1.07)	0.87 (0.79, 0.96)	0.91 (0.83, 1.00)	0.008
P-value	0.214		0.531	0.005	0.059	
300m buffer	0.96 (0.90, 1.02)	Ref.	0.97 (0.88, 1.07)	0.95 (0.86, 1.05)	0.93 (0.85, 1.02)	0.054
P-value	0.216		0.559	0.317	0.141	

Models were adjusted for age, sex, body mass index, ethnicity, Townsend deprivation index, smoking status, alcohol intake, physical activity, living score, lymphocyte percentage, white blood cell count, and C-reactive protein.
Abbreviations: CI, confidence interval; Q, quantile.

Table S5. Associations of residential natural environment, domestic garden, green space, and blue space with the risk of incident psoriasis after further adjusting for cancer, diabetes, and cardiovascular disease.

	Hazard ratio (95% CI)					*P* for trend
	Continuous	Q1	Q2	Q3	Q4	
Case/total			3755/385145			
Natural environment						
1000m buffer	1.05 (1.00, 1.11)	Ref.	1.11 (1.01, 1.22)	1.09 (0.98, 1.21)	1.16 (1.04, 1.29)	0.018
P-value	0.039		0.032	0.100	0.007	
300m buffer	1.03 (1.01, 1.06)	Ref.	1.05 (0.95, 1.15)	1.06 (0.96, 1.17)	1.12 (1.02, 1.24)	0.024
P-value	0.016		0.360	0.234	0.022	
Domestic garden						
1000m buffer	0.93 (0.89, 0.98)	Ref.	0.93 (0.85, 1.02)	0.99 (0.91, 1.08)	0.85 (0.77, 0.93)	0.005
P-value	0.009		0.124	0.840	0.001	
300m buffer	0.94 (0.90, 0.99)	Ref.	1.00 (0.91, 1.09)	0.98 (0.89, 1.07)	0.91 (0.83, 1.00)	0.046
P-value	0.012		0.936	0.599	0.046	
Green space						
1000m buffer	1.07 (1.00, 1.16)	Ref.	1.03 (0.93, 1.13)	1.08 (0.98, 1.19)	1.15 (1.04, 1.28)	0.004
P-value	0.063		0.616	0.142	0.007	
300m buffer	1.04 (0.99, 1.09)	Ref.	0.97 (0.89, 1.07)	1.02 (0.93, 1.12)	1.09 (0.99, 1.20)	0.041
P-value	0.132		0.550	0.630	0.072	
Blue space						
1000m buffer	0.97 (0.91, 1.02)	Ref.	0.98 (0.89, 1.07)	0.89 (0.81, 0.97)	0.93 (0.85, 1.02)	0.030
P-value	0.221		0.613	0.011	0.110	
300m buffer	0.96 (0.91, 1.02)	Ref.	0.96 (0.88, 1.05)	0.94 (0.86, 1.03)	0.93 (0.85, 1.02)	0.097
P-value	0.222		0.401	0.171	0.115	

Models were adjusted for age, sex, body mass index, ethnicity, Townsend deprivation index, smoking status, alcohol intake, physical activity, living score, cancer, diabetes, and cardiovascular disease.

Abbreviations: CI, confidence interval; Q, quantile.

Table S6. Associations of residential natural environment, domestic garden, green space, and blue space with the risk of incident psoriasis after restricting participants living at the same address >3 years.

	Hazard ratio (95% CI)					*P* for trend
	Continuous	Q1	Q2	Q3	Q4	
Case/total			3450/355053			
Natural environment						
1000m buffer	1.05 (1.00, 1.10)	Ref.	1.11 (1.01, 1.23)	1.09 (0.98, 1.21)	1.15 (1.03, 1.28)	0.033
P-value	0.066		0.035	0.122	0.013	
300m buffer	1.03 (1.01, 1.06)	Ref.	1.06 (0.96, 1.16)	1.06 (0.96, 1.18)	1.11 (1.00, 1.23)	0.050
P-value	0.018		0.278	0.222	0.048	
Domestic garden						
1000m buffer	0.94 (0.89, 0.99)	Ref.	0.94 (0.85, 1.03)	1.01 (0.92, 1.11)	0.84 (0.77, 0.93)	0.007
P-value	0.019		0.164	0.858	<0.001	
300m buffer	0.95 (0.90, 0.99)	Ref.	1.01 (0.92, 1.11)	0.97 (0.89, 1.07)	0.90 (0.82, 0.99)	0.029
P-value	0.021		0.845	0.571	0.038	
Green space						
1000m buffer	1.07 (0.99, 1.16)	Ref.	1.03 (0.93, 1.14)	1.08 (0.97, 1.20)	1.15 (1.04, 1.28)	0.006
P-value	0.082		0.538	0.142	0.010	
300m buffer	1.04 (0.99, 1.09)	Ref.	0.98 (0.89, 1.07)	1.03 (0.93, 1.13)	1.09 (0.99, 1.21)	0.052
P-value	0.154		0.612	0.606	0.087	
Blue space						
1000m buffer	0.96 (0.90, 1.02)	Ref.	0.99 (0.90, 1.08)	0.89 (0.81, 0.98)	0.93 (0.84, 1.02)	0.027
P-value	0.148		0.780	0.014	0.108	
300m buffer	0.96 (0.90, 1.02)	Ref.	0.93 (0.84, 1.02)	0.92 (0.84, 1.01)	0.92 (0.83, 1.01)	0.083
P-value	0.217		0.113	0.090	0.073	

Models were adjusted for age, sex, body mass index, ethnicity, Townsend deprivation index, smoking status, alcohol intake, physical activity, and living score.

Abbreviations: CI, confidence interval; Q, quantile.

Table S7. Associations of residential natural environment, domestic garden, green space, and blue space with the risk of incident psoriasis with the imputation of covariates.

	Hazard ratio (95% CI)					*P* for trend
	Continuous	Q1	Q2	Q3	Q4	
Case/total	4227/431412					
Natural environment						
1000m buffer	1.05 (1.01, 1.10)	Ref.	1.10 (1.00, 1.20)	1.10 (1.00, 1.21)	1.12 (1.02, 1.24)	0.037
P-value	0.031		0.047	0.049	0.023	
300m buffer	1.03 (1.00, 1.05)	Ref.	1.04 (0.95, 1.13)	1.06 (0.97, 1.16)	1.08 (0.99, 1.19)	0.081
P-value	0.033		0.393	0.176	0.092	
Domestic garden						
1000m buffer	0.95 (0.91, 1.00)	Ref.	0.96 (0.89, 1.05)	1.02 (0.94, 1.11)	0.87 (0.80, 0.95)	0.018
P-value	0.051		0.391	0.684	0.003	
300m buffer	0.96 (0.93, 1.01)	Ref.	1.02 (0.94, 1.11)	0.99 (0.91, 1.08)	0.95 (0.87, 1.03)	0.173
P-value	0.109		0.649	0.826	0.209	
Green space						
1000m buffer	1.07 (1.00, 1.15)	Ref.	1.03 (0.94, 1.13)	1.11 (1.01, 1.22)	1.12 (1.02, 1.24)	0.009
P-value	0.063		0.527	0.030	0.022	
300m buffer	1.03 (0.99, 1.08)	Ref.	0.99 (0.91, 1.08)	1.04 (0.95, 1.13)	1.08 (0.99, 1.18)	0.058
P-value	0.160		0.855	0.430	0.091	
Blue space						
1000m buffer	0.96 (0.91, 1.01)	Ref.	0.97 (0.89, 1.05)	0.90 (0.83, 0.98)	0.92 (0.84, 1.00)	0.015
P-value	0.105		0.455	0.016	0.045	
300m buffer	0.94 (0.89, 1.00)	Ref.	0.95 (0.87, 1.03)	0.93 (0.85, 1.02)	0.90 (0.83, 0.99)	0.021
P-value	0.042		0.209	0.104	0.021	

Models were adjusted for age, sex, body mass index, ethnicity, Townsend deprivation index, smoking status, alcohol intake, physical activity, and living score.

Abbreviations: CI, confidence interval; Q, quantile.

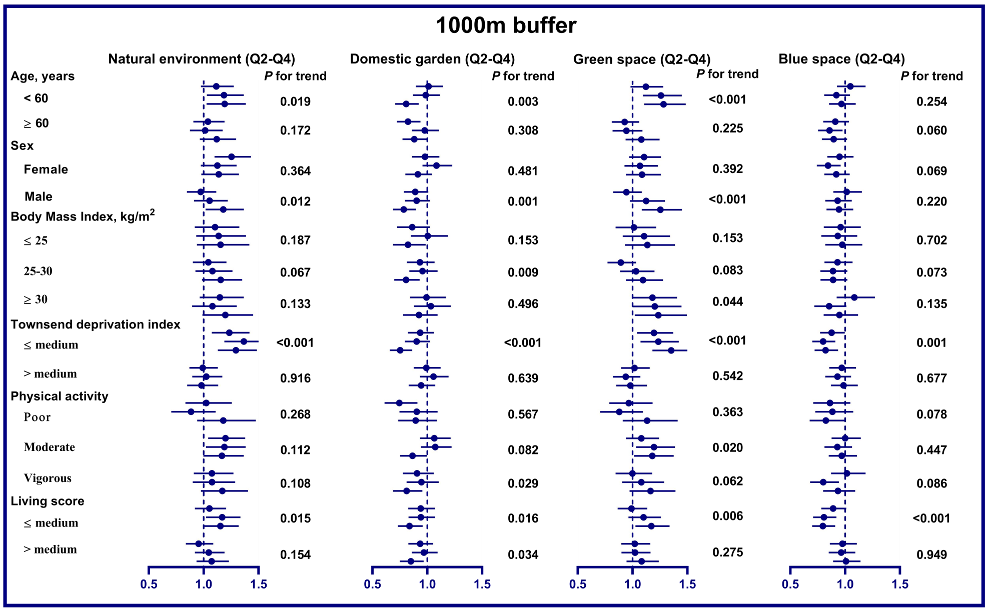

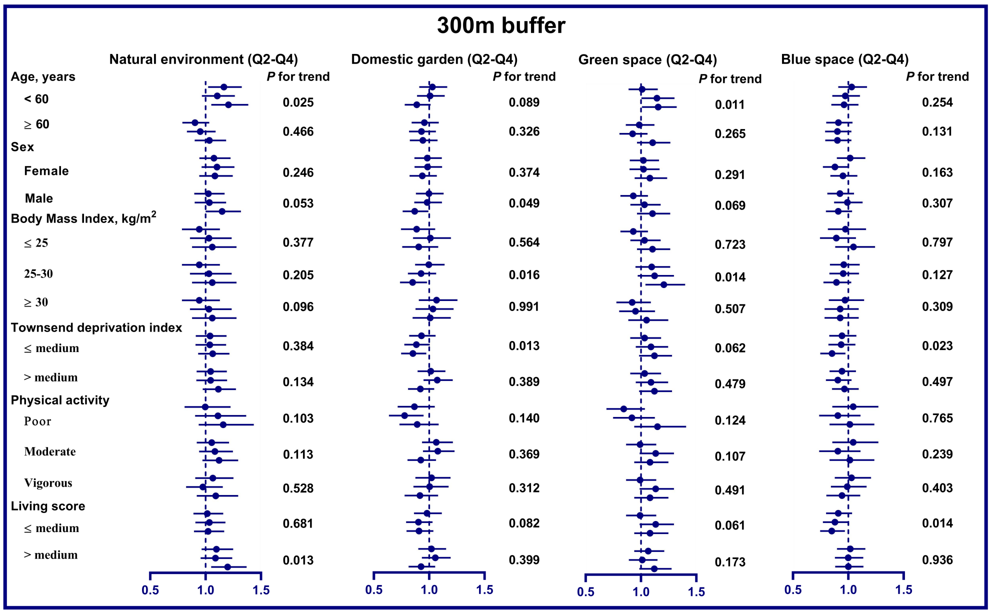

Figure S5. Stratified analyses of the associations of residential natural environment, domestic garden, green space, and blue space with incident psoriasis. Models were adjusted for age, sex, body mass index, ethnicity, Townsend deprivation index, smoking status, alcohol intake, physical activity, and living score. Abbreviations: Q, quantile.

Table S8. Additive and multiplicative interactions of residential natural environment and domestic garden, green space and blue space on the risk of incident psoriasis.

	RERI (95% CI)	AP (95% CI)	*P*-value additive interactions	*P*-value multiplicative interactions
Natural environment-Domestic garden				
1000m buffer	0.01 (-0.04, 0.05)	0.01 (-0.03, 0.04)	0.771	0.730
300m buffer	-0.03 (-0.08, 0.03)	-0.02 (-0.05, 0.00)	0.348	0.480
Green space-Blue space				
1000m buffer	-0.06 (-0.11, -0.01)	-0.06 (-0.03, -2.35)	0.019	0.022
300m buffer	-0.02 (-0.07, 0.04)	-0.02 (-0.05, 0.02)	0.537	0.560

Models were adjusted for age, sex, body mass index, ethnicity, smoking status, alcohol intake, Townsend deprivation index, physical activity, and living score,.

Abbreviations: RERI, relative excess risk due to interaction; AP, attributable proportion due to interaction; CI, confidence interval.

Table S9. Summary results of SNPs used for PRS in the study.

Chromosome	rsID	Effect allele	Position	Ln (OR)	*P*
1	rs7524364	A	8286009	0.11	2.34E-07
1	rs10794648	T	24518206	-0.18	1.02E-17
1	rs113935720	T	67713346	0.36	6.08E-21
1	rs10789285	T	69788482	-0.07	8.35E-04
1	rs34517439	A	78450517	0.17	1.19E-06
1	rs4845453	C	152591953	0.18	3.70E-23
1	rs12118303	T	172675097	-0.11	2.07E-07
1	rs17022427	C	206654497	0.1	5.91E-07
2	rs4672505	A	62560332	0.11	7.65E-10
2	rs17715343	C	163167746	0.31	7.1-E-20
3	rs13080782	A	16996623	-0.12	7.82E-12
3	rs1707602	T	101647309	-0.1	1.13E-08
3	rs28512356	C	189615475	0.1	2.31E-04
5	rs112768831	A	40370724	-0.12	2.43E-04
5	rs27044	C	96118852	-0.15	5.63E-16
5	rs1295685	A	131996445	-0.18	1.53E-16
5	rs74817271	A	150469973	0.48	2.73E-49
5	rs12188300	A	158829527	-0.51	1.82E-69
6	rs7748720	A	20689945	0.16	2.67E-14
6	rs13200483	A	30916259	1.15	1.60E-299
6	rs582757	T	138197824	-0.18	6.55E-22
7	rs11767350	A	37385365	0.1	5.55E-09
9	rs11795343	T	32523737	0.11	2.04E-10
9	rs10816610	A	110781922	0.1	1.72E-07
10	rs2944542	C	64369999	-0.08	1.35E-05
10	rs1108618	A	81043743	0.11	3.56E-10
10	rs76959677	A	89824771	-0.25	1.37E-06
11	rs2510066	C	64052447	0.1	1.07E-08
11	rs4561177	A	109962432	0.13	5.97E-15
11	rs61907765	T	128391937	0.13	4.44E-10
12	rs57137641	A	56741228	-0.35	1.42E-21
12	rs11059675	A	122668326	0.09	2.77E-06
13	rs73183592	A	40745693	-0.2	4.59E-06
13	rs4942358	A	45321731	-0.1	5.22E-07
14	rs2145623	C	35839236	0.15	4.11E-16
14	rs79470265	C	98649129	-0.12	5.87E-07
15	rs28624578	T	31637666	0.16	8.26E-09
16	rs413024	A	11354091	0.12	4.85E-11
17	rs28998802	A	26124908	0.22	3.91E-20
17	rs8070763	T	40536396	-0.11	3.01E-09
17	rs55823223	A	73890363	0.13	8.39E-08
17	rs2304856	T	78175483	0.08	2.31E-04

19	rs34536443	C	10463118	-0.68	3.49E-35
19	rs4804528	T	10886206	-0.11	1.40E-09
21	rs17812953	T	36488822	-0.1	5.40E-05

Abbreviations: SNP, single nucleotide polymorphism; OR, odds ratio;; PRS, polygenic risk score.

Table S10. Hazard ratio of psoriasis risk based on PRS.

	PRS Hazard ratio (95% CI)	*P*-value
	2379/240558	
Continuous	1.38 (1.31, 1.45)	<0.001
Low genetic risk	1 (Reference)	
Medium genetic risk	1.44 (1.28, 1.62)	<0.001
High genetic risk	2.05 (1.79, 2.34)	<0.001
P for trend	<0.001	

Models were adjusted for age, sex, body mass index, ethnicity, smoking status, alcohol intake, Townsend deprivation index, physical activity, living score, genotyping batch, and genetic principal components.

Abbreviations: PRS, polygenic risk score; CI, confidence interval.

Table S11. Additive and multiplicative interactions of residential natural environment, domestic garden, green space, and blue space with PRS on the risk of incident psoriasis.

	RERI (95% CI)	AP (95% CI)	*P*-value additive interactions	*P*-value multiplicative interactions
Natural environment				
1000m buffer	0.01 (-0.06, 0.08)	0.01 (-0.02, 0.04)	0.781	0.913
300m buffer	0.03 (-0.01, 0.07)	0.02 (0.01, 0.04)	0.197	0.215
Domestic garden				
1000m buffer	-0.05 (-0.16, 0.05)	-0.04 (-0.06, -0.01)	0.314	0.619
300m buffer	-0.03 (-0.15, 0.08)	-0.02 (-0.05, 0.01)	0.588	0.240
Green space				
1000m buffer	0.01 (-0.10, 0.11)	0.01 (-0.03, 0.04)	0.896	0.532
300m buffer	0.04 (-0.04, 0.11)	0.03 (0.01, 0.06)	0.329	0.385
Blue space				
1000m buffer	-0.16 (-0.28, -0.04)	-0.12 (0.19, -0.04)	0.011	0.061
300m buffer	-0.10 (-0.21, 0.02)	-0.07 (-0.15, 0.01)	0.103	0.347

Models were adjusted for age, sex, body mass index, ethnicity, smoking status, alcohol intake, Townsend deprivation index, physical activity, living score, genotyping batch, and genetic principal components.

Abbreviations: PRS, polygenic risk score; RERI, relative excess risk due to interaction; AP, attributable proportion due to interaction; CI, confidence interval.

Table S12. Hazard ratio of psoriasis risk based on Standard PRS.

	PRS<br>Hazard ratio (95% CI)	*P*-value
	3642/374741	
Continuous	1.33 (1.29, 1.37)	<0.001
Low genetic risk	1 (Reference)	
Medium genetic risk	1.41 (1.28, 1.56)	<0.001
High genetic risk	2.29 (2.05, 2.55)	<0.001
P for trend	<0.001	

Models were adjusted for age, sex, body mass index, ethnicity, smoking status, alcohol intake, Townsend deprivation index, physical activity, living score, genotyping batch, and genetic principal components.

Abbreviations: PRS, polygenic risk score; CI, confidence interval.

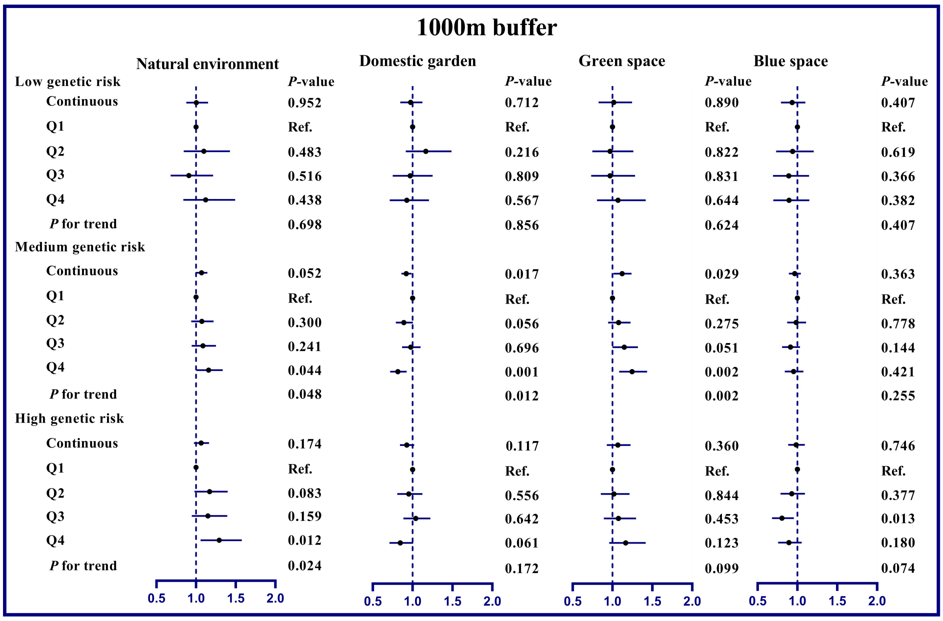

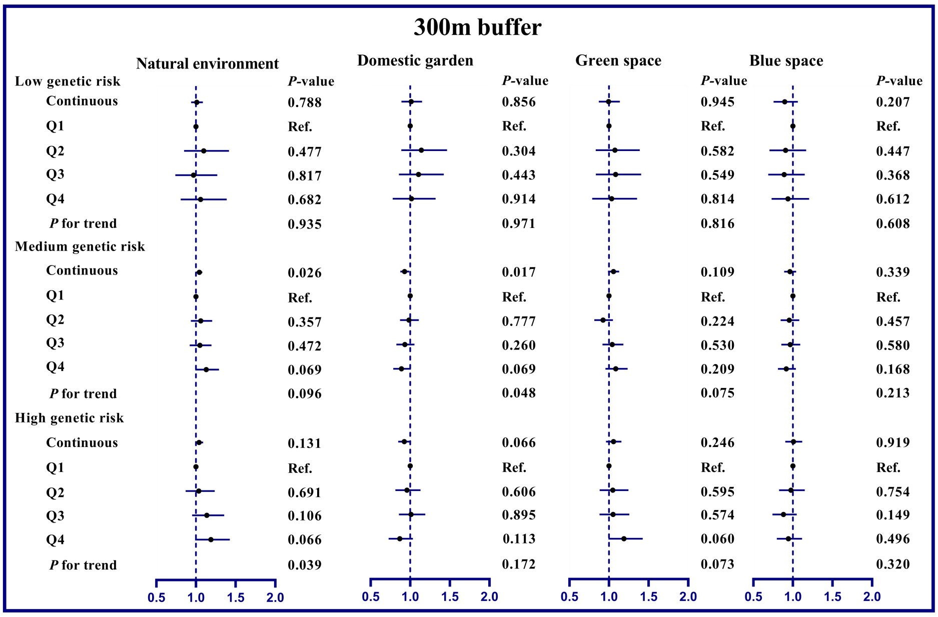

Figure S6. Hazard ratio of psoriasis risk based on residential natural environment, domestic garden, green space and blue space stratified by Standard PRS. Models were adjusted for age, sex, body mass index, ethnicity, smoking status, alcohol intake, Townsend deprivation index, physical activity, living score, genotyping batch, and genetic principal components.

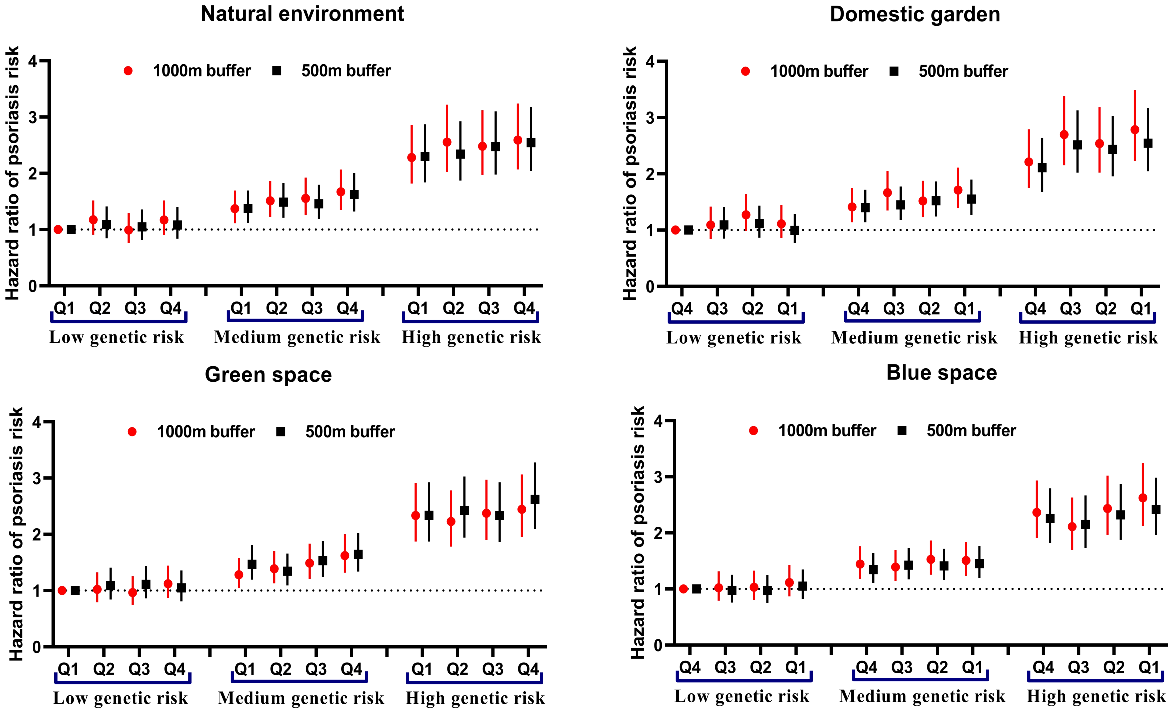

Figure S7. Joint effects of residential natural environment, domestic garden, green space and blue space with PRS on the risk of psoriasis risk. Models were adjusted for age, sex, body mass index, ethnicity, smoking status, alcohol intake, Townsend deprivation index, physical activity, living score, genotyping batch, and genetic principal components. Abbreviations: RA, rheumatoid arthritis; PRS, polygenic risk score, Q, quantile.

Table S13. Additive and multiplicative interactions of residential natural environment, domestic garden, green space, and blue space with Standard PRS on the risk of incident psoriasis.

	RERI (95% CI)	AP (95% CI)	*P*-value additive interactions	*P*-value multiplicative interactions
Natural environment				
1000m buffer	-0.01 (-0.07, 0.04)	-0.01 (-0.03, 0.01)	0.630	0.187
300m buffer	-0.01 (-0.04, 0.03)	0.00 (-0.02, 0.01)	0.740	0.284
Domestic garden				
1000m buffer	0.00 (-0.06, 0.07)	0.00 (-0.02, 0.03)	0.921	0.434
300m buffer	-0.03 (0.09, 0.04)	-0.02 (-0.04, 0.00)	0.426	0.848
Green space				
1000m buffer	-0.02 (-0.11, 0.07)	-0.01 (-0.04, 0.02)	0.636	0.111
300m buffer	0.00 (-0.05, 0.06)	0.00 (-0.02, 0.02)	0.981	0.570
Blue space				
1000m buffer	-0.04 (-0.07, 0.00)	-0.03 (-0.05, -0.01)	0.050	0.185
300m buffer	0.00 (-0.07, 0.08)	0.00 (-0.05, 0.06)	0.891	0.561

Models were adjusted for age, sex, body mass index, ethnicity, smoking status, alcohol intake, Townsend deprivation index, physical activity, living score, genotyping batch, and genetic principal components.

Abbreviations: PRS, polygenic risk score; RERI, relative excess risk due to interaction; AP, attributable proportion due to interaction; CI, confidence interval.
